# A Novel Cellulase Produced by a Newly Isolated *Trichoderma virens*

**DOI:** 10.3390/bioengineering3020013

**Published:** 2016-04-19

**Authors:** Rong Zeng, Xiao-Yan Yin, Tao Ruan, Qiao Hu, Ya-Li Hou, Zhen-Yu Zuo, Hao Huang, Zhong-Hua Yang

**Affiliations:** 1 College of Chemistry and Chemical Engineering, Hubei University, Wuhan 430062, China; rongzengce@163.com; 2 College of Chemical Engineering and Technology, Wuhan University of Science and Technology, Wuhan 430081, China; yinxiaoyan0516@163.com (X.-Y.Y.); 15671627439@163.com (T.R.); huqiao294873270@163.com (Q.H.); houyali88@163.com (Y.-L.H.); zuozhenyu@wust.edu.cn (Z.-Y.Z.); hhzy310@163.com (H.H.)

**Keywords:** *Trichoderma virens*, cellulase, strain isolation, straw, biomass

## Abstract

Screening and obtaining a novel high activity cellulase and its producing microbe strain is the most important and essential way to improve the utilization of crop straw. In this paper, we devoted our efforts to isolating a novel microbe strain which could produce high activity cellulase. A novel strain *Trichoderma virens* ZY-01 was isolated from a cropland where straw is rich and decomposed, by using the soil dilution plate method with cellulose and Congo red. The strain has been licensed with a patent numbered ZL 201210295819.6. The cellulase activity in the cultivation broth could reach up to 7.4 IU/mL at a non-optimized fermentation condition with the newly isolated *T. virens* ZY-01. The cellulase was separated and purified from the *T. virens* culture broth through (NH_4_)_2_SO_4_ fractional precipitation, anion-exchange chromatography and gel filtration chromatography. With the separation process, the CMC specific activity increased from 0.88 IU/mg to 31.5 IU/mg with 35.8 purification fold and 47.04% yield. Furthermore, the enzymatic properties of the cellulase were investigated. The optimum temperature and pH is 50 °C and pH 5.0 and it has good thermal stability. Zn^2+^, Ca^2+^ and Mn^2+^ could remarkably promote the enzyme activity. Conversely, Cu^2+^ and Co^2+^ could inhibit the enzymatic activity. This work provides a new highly efficient *T. virens* strain for cellulase production and shows good prospects in practical application.

## 1. Introduction

With the development of agriculture, cellulose-rich straws from cropland have become one of the largest amounts of biomass. They should be comprehensively utilized as biological resources. Unfortunately, most crop straws, such as wheat straw and rice straw, are directly burned in cropland, which has caused serious atmospheric pollution in China [1]. If biological resources that are prevented from being directly burned in cropland can be utilized as carbohydrates, then the atmospheric pollution can be avoided and a huge amount of carbohydrates, such as fermentable sugars, can be provided. It is well known that the main component of straw is cellulose, which is the most common source of renewable carbon and energy on earth. Cellulose could be efficiently degraded to fermentable sugars with conventional biological processes. The fermentable sugars are the most important platform material, which could be further converted to ethanol (biofuel), single cell protein (SCP) and other chemicals by biotechnology [2,3,4]. In the process route, a high activity of cellulase is the key factor to this biological process of straw utilization as biological resources [5,6,7]. Screening and obtaining a novel high activity cellulase and its producing microbe strain is always the most important and essential way to improve the straw utilization. Moreover, cellulase has a very broad application in other fields such as food processing, oil extraction, agricultural industries, brewery, animal feed, textile and other fields like laundry, pulp, paper, detergent industry [8,9,10,11,12]. 

Cellulase is mainly produced by microbes, especially fungus, such as *Trichoderma*, *Aspergillus* and *Penicillium*. Nowadays, more and more *Trichoderma* fungi have been screened; however, there exists a lower-yield problem, so it is important to screen a strain that produces cellulase efficiently.

In this paper, we devoted our efforts to isolate a novel microbe strain which can produce high activity cellulase. Furthermore, the enzymatic properties of this new cellulase will also be evaluated.

## 2. Materials and Methods

### 2.1. Material and Media

The soil samples were obtained from land areas where straw is rich and some straw was decomposed. We collected soil samples from a wheat field (33°36′41″N and 116°56′17″E, Suzhou county, Anhui province, China), Yangtze River riverbank (30°38′14″N and 114°22′4″E, Wuhan City, Hubei province, China), and a paper mill (30°27′3″N, 114°11′59″E, Wuhan City, Hubei province, China).

The following mediums were used for enrichment, screening, identification and cultivation process. the enrichment medium was used to enrich the microbe, and it was composed of (in g/L): oatmeal extract (oatmeal 3.5 g adding water 30 mL and boil 20–30 min then filter to obtain the filtrate), agar 1.5, crystal violet 0.5, deionized water 100 mL. The Congo red medium was used to screen the target strain as the screening medium, it was composed of (in g/L): KH_2_PO_4_ 1.0, NaNO_3_ 3.0, KCl 0.5, MgSO_4_·7H_2_O 0.5, FeSO_4_·7H_2_O 0.01, CMC∙Na 15, Agar 15–20 and Congo red 0.2. The identification medium was used to identify the target strain, which was composed of (in g/L): NaNO_3_ 3.0, MgSO_4_ 0.5, K_2_HPO_4_ 1.0, KCl 0.5, FeSO_4_ 0.01, sucrose or glucose 30.0. The fermentation medium was used to culture the target strain and produce cellulase, it was composed of (in g/L): yeast extract 1, CMC∙Na 5, NaCl 20, K_2_HPO_4_ 0.5 and MgSO_4_∙7H_2_O 0.5.

### 2.2. Screening the Microbe for Cellulase and Microbe Identification

The soil sample (2.0 g) was suspended in 20 mL of sterile distilled water. Then 0.5 mL supernatant was inoculated on enrichment medium plates with coating inoculation, and cultured for three days at 30 °C. The colonies growing in the enrichment medium plates were inoculated in the Congo red medium plate with inoculating needle and further cultured on the third day at 30 °C. The colonies growing on the Congo red plate were the microbes which can excrete cellulase. The diameters of the colony and hydrolyzed circle can indicate the microbe’s ability to produce cellulase [10]. The selected colonies were further purified by culture in new Congo red medium plate.

Morphological and molecular identification of the isolated microbes were done according to the Fungal Identification Manual [13]. The identification medium was used to culture the screened strain. A light microscope was used to observe its morphological features. Genomic DNA for molecular identification of the strain was extracted using the modified benzyl chloride method [14]. In order to identify the isolated microbe, the 18S rDNA was cloned by PCR (Initialization at 94 °C for 5 min, Denaturation at 94 °C for 30 s, Annealing at 54 °C for 1 min, Extension at 72 °C for 90 s, 30 cycles, Final elongation at 72 °C for 10 min, 4 °C to the end, PCR in 25 μL reaction system with pfu DNA polymerase) with the primers (Forward: 5′-GTAGTCATATGCTTGTCTC-3′, Reverse: 5′-TCCGCAGGTTCACCTACGGA-3′). The PCR-amplified products were purified by Gel Extraction kit (Omega Bio-Tek, Norcross, GA, USA) and sequenced by Sangon Biotech (Shanghai, China). The 18S rDNA was compared with sequences in nucleotide database (NCBI) using the BLAST algorithm. Multiple sequence alignment was carried out with CLUSTALW (Conway Institute UCD, Dublin, Ireland) multiple sequence alignment. The neighbour-joining phylogenetic analysis was constructed with MEGA v.5.0 software (Center for Evolutionary Medicine and Informatics, The Biodesign Institute, Tempe, AZ, USA).

### 2.3. Purification of the Cellulase from the Newly Isolated Microbe Culture Broth

The cellulase was produced by the newly isolated strain with fermentation medium at 30 °C for 4 days. The crude enzyme extraction was collected by centrifuging the cultivation broth, since cellulase is an extracellular enzyme. It was further purified by fractionation salting out (50%–70% saturation of ammonium sulfate), anion exchange chromatography (DEAE Sepharose Fast Flow, elution with 0–0.8 M·NaCl) and gel filtration chromatography (SephadexG-75). After purification, the purified product was assayed by SDS-PAGE.

### 2.4. Evaluation of Enzymatic Properties

The following enzymatic properties of the cellulase produced by the newly isolated microbe were evaluated. The optimum pH and pH stability, the optimum temperature and thermal stability and effects of metal ions to the enzymatic activity were investigated. Er mixed 0.5 mL enzyme with 0.5 mL substrate containing 0.05% (*w*/*v*) CMC-Na in citric acid buffer and incubated the mixture (pH = 5.0) for 1 h. The temperature range was from 30 °C to 90 °C, and pH was range from 2 to 9, and the concentration of metal ions was 0.01 mol/L. After incubation, we measured the cellulase activity, as described below.

### 2.5. Cellulase Activity Assay

The activity of the cellulase was assayed with CMC-Na activity measurement [15]. An enzyme sample of 0.5 mL was added into 1.5 mL of the reaction mixture (containing 0.05% (*w*/*v*) CMC∙Na with pH 5.0 citric acid buffer) and incubated at 50 °C for 1 h. Then the reducing sugar released by the reaction was determined by DNS method. One unit of the CMC enzyme activity was defined as the amount of enzyme that catalyzed to produce 1 μmol of reduced sugar per minute with the reduction of CMC-Na.

## 3. Results

### 3.1. Isolation of Microbe for Cellulase and Microbe Identification

The purpose of isolation was to obtain a microbial strain that possessed ability to produce high activity cellulase. With the isolation procedure, four microbial strains were obtained and tested for cellulase activity, which can be released from cellulase to hydrolysis the CMC∙Na and formed an obvious hydrolyzed circle. Table 1 describes the physiological characteristics of the isolated strains.

In terms of the diameter size of the colony and hydrolysised circle that appeared on the plates, strain ZY-01 is the best strain for cellulase production. Since the strain possesses more cellulase production ability, then more cellulase activity can be released to hydrolysis of the CMC-Na and a larger hydrolysised circle and colony were formed. It can be confirmed that ZY-01 is a good high-producing strain. The cellulase production ability of this newly isolated ZY-01 strain was tested. The cellulase activity 7.4 IU/mL was obtained at a non-optimized fermentation condition.

**Figure 1:**
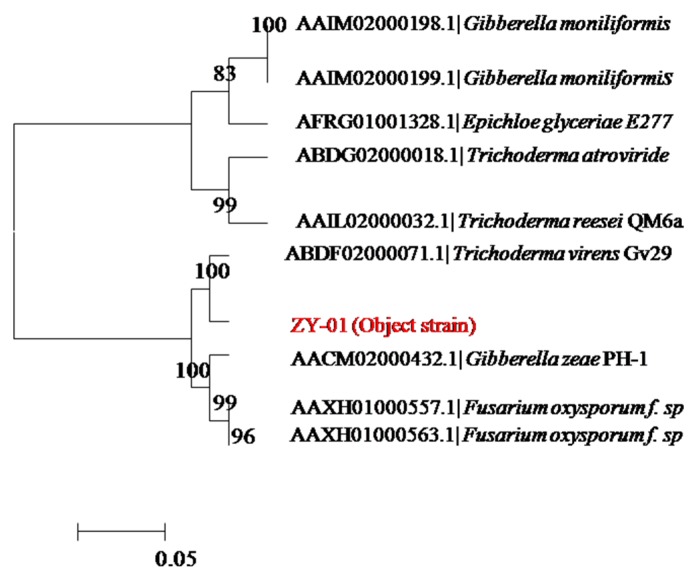
Neighbor-joining phylogenetic tree of 18S rDNA genes of strain ZY-01.

The colony morphology and the morphological features of mycelium by light microscope both indicated that the screened ZY-01was a fungi. To further identify strain ZY-01, its 18S rDNA region was cloned by PCR and sequenced. Its nucleotide blast analysis was conducted in NCBI to find homologous sequences. The neighbor-joining phylogenetic tree, Figure 1, was constructed with these homologous sequences. The results show that strain ZY-01 is a *Trichoderma virens*. Furthermore, the morphological identification experiments also confirm that it is a *T. virens* according to the Fungal Identification Manual [13]. Then this newly isolated strain was named T. virens ZY-01 and deposited in the China Center for Type Culture Collection (CCTCC) with an accession number CCTCC M 2012205. Its 18S rDNA sequence was deposited at the GenBank database with the Accession No. JX121089. The cellulase productivity of this newly isolated *T. virens* is appealing compared to the previous reports [11,16,17,18]. According to these reports, the cellulase activity was about 7.8, 0.18, 1.43 and 7.51 IU/mL to the corresponding *T. reesei*, Mutated *T. viride*, *Fusarium oxysporum* H57-1 and *Rhizopus stolonifer* var. reflexus TP-02 at the optimized enzyme production conditions.

### 3.2. Purification of the Cellulase from the Newly Isolated T. virens ZY-01 Culture Broth

**Figure 2:**
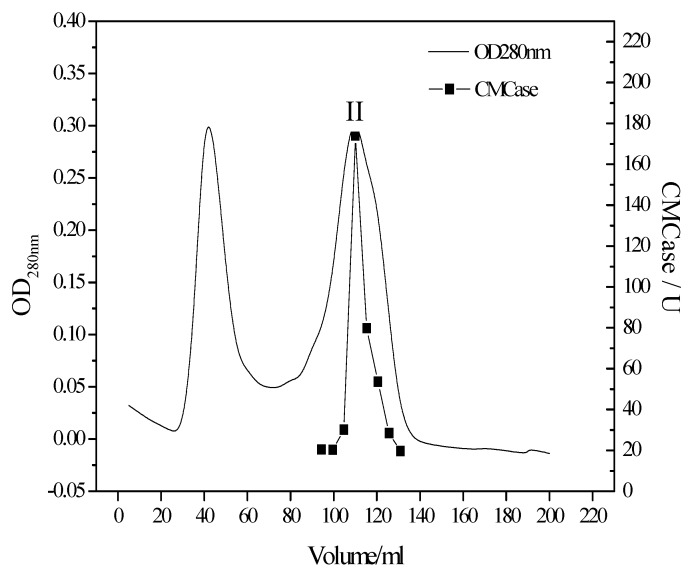
Elution profile gel filtration column chromatography.

The cellulase was purified from the cultivation broth of *T. virens* ZY-01. With three complementary purification steps (fractional precipitation, anion exchange chromatography and gel filtration chromatography) Sephadex G-75 chromatography elution was distinguished by molecular weight of protein. The elution curves are shown in Figure 2. The CMCase activity mainly assembled at peak II.

As a result, cellulase was purified 35.8-fold with a 47.04% CMC enzymatic activity yield compared to the crude enzyme. The results of each separation step are given in Table 2.

**Figure 3:**
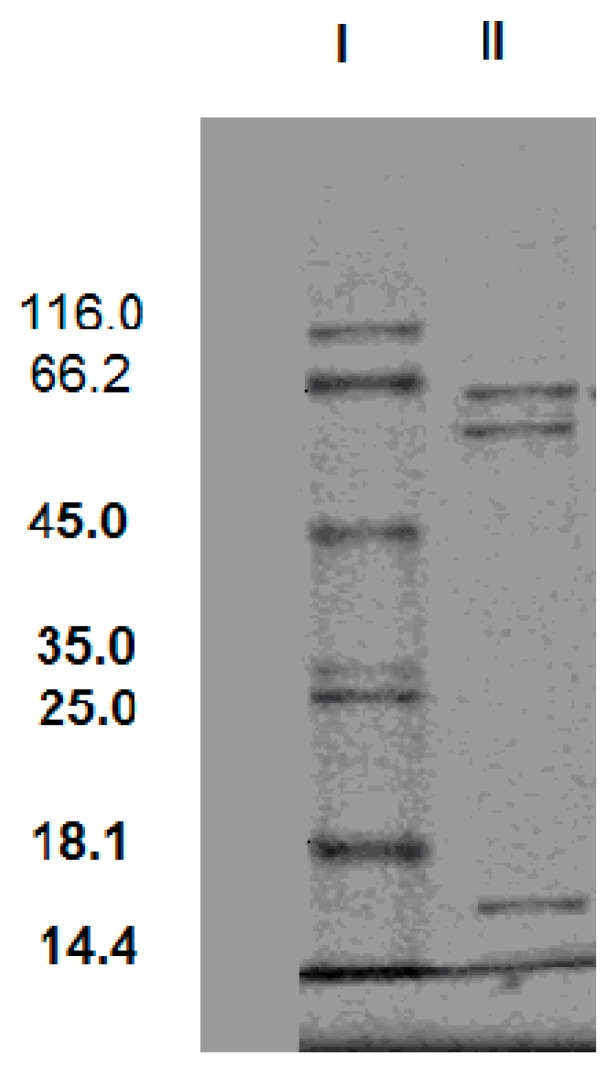
SDS-PAGE of the purification product from the *T. virens* ZY-01 cultivation broth. Lane I: protein ladder (β-galactosidase 116.0 kDa, bovine serum albumin 66.2 kDa, ovalbumin 45.0 kDa, lactate dehydrogenase 35.0 kDa, REase Bsp981 25.0 kDa, β-lactoglobulin 18.1 kDa, lysozyme 14.4 kDa), Lane II: purification product.

The SDS-PAGE of the purification product was given in Figure 3. The SDS-PAGE result showed that there were three protein bands with molecular masses of 62 kDa, 58 kDa and 16 kDa. It indicates that this cellulase is a triple-subunit complex. In general, cellulase is multi-subunit complex. Tang and Balasubramanian respectively reported cellulases from *Rhizopus stolonifer* var. reflexus TP-02O and *Bacillus mycoides* S122C also had three subunits [17,18]. The molecular masses of the subunit are from 40 kDa to 64 kDa.

### 3.3. Enzymatic Properties of Cellulase Produced by T. virens ZY-01

**Figure 4:**
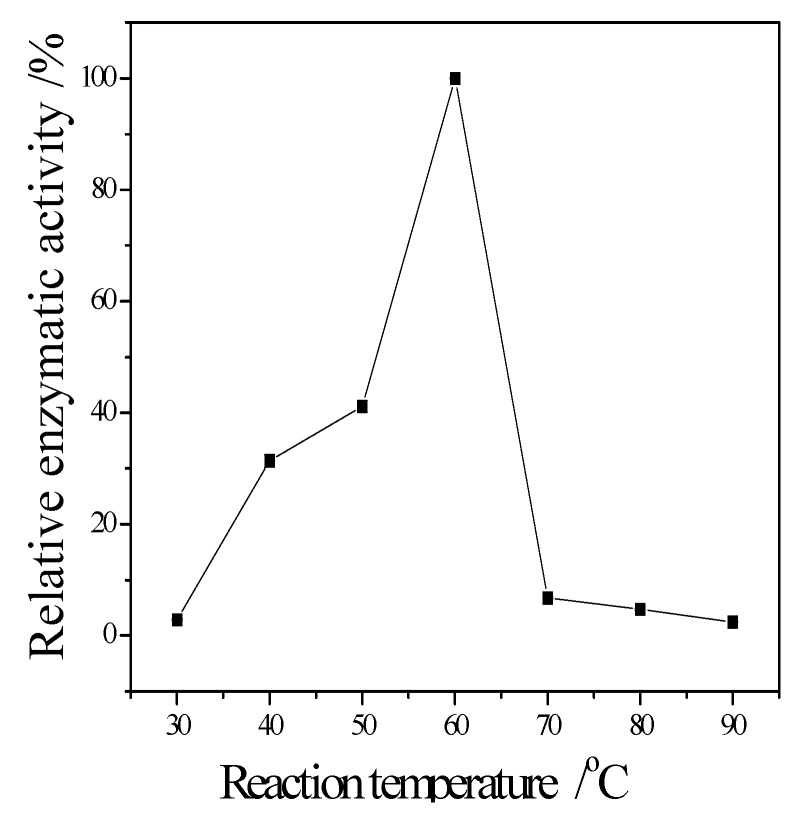
Effect of reaction temperature on enzymatic activity.

**Figure 5:**
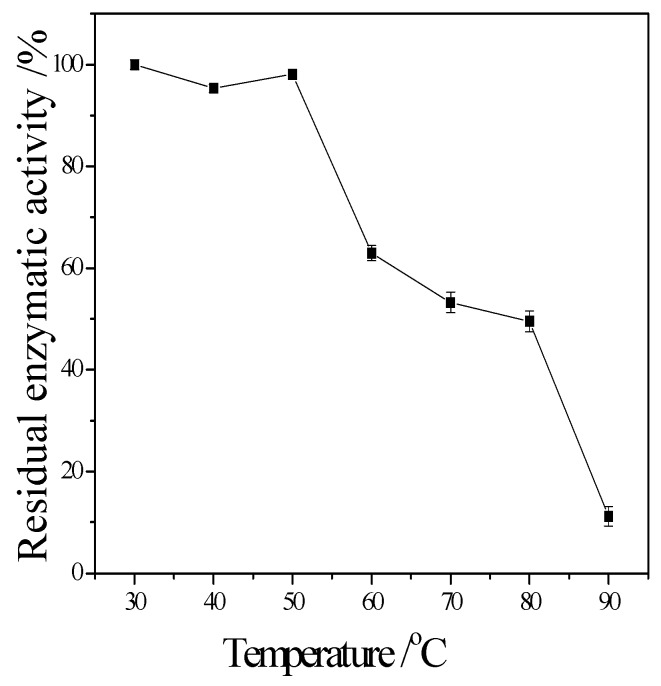
Thermal stability of the cellulose.

**Figure 6:**
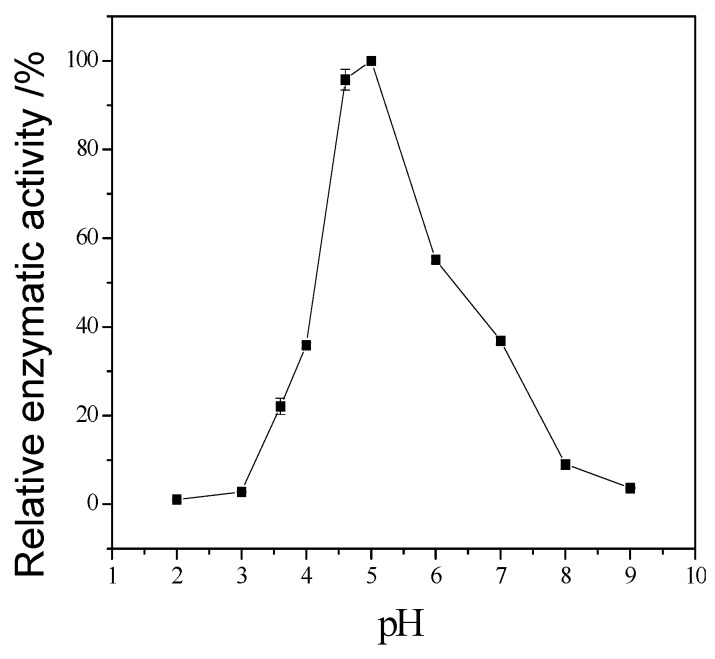
Effect of reaction pH on enzymatic activity.

**Figure 7:**
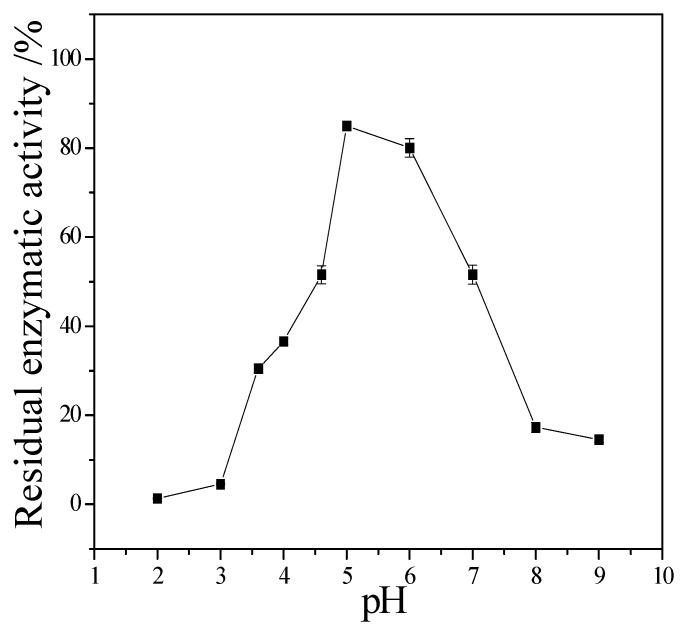
Stability of the cellulase to pH.

**Figure 8:**
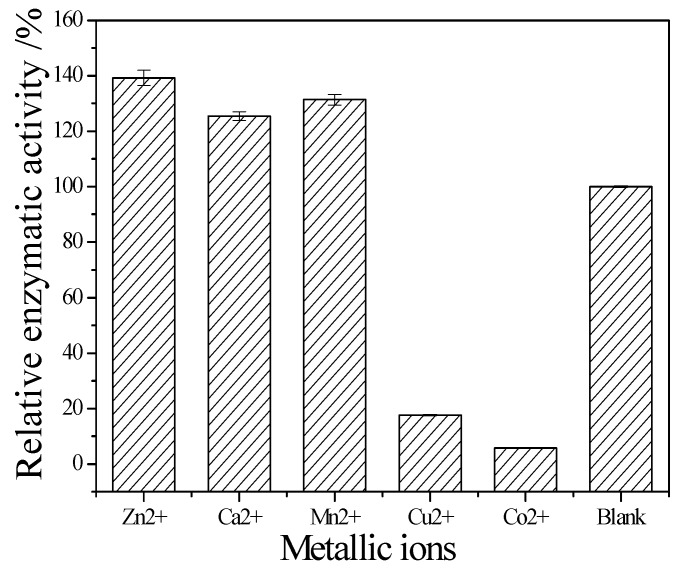
Effects of metal ions on enzymatic activity.

The enzymatic properties are the fundamental bioinformation to an enzyme. They are important to it application. The following enzymatic properties of the new cellulase produced by *T. virens* ZY-01 were evaluated: the effect of reaction temperature, pH and metal ions to enzymatic activity, the thermal stability and pH stability. The corresponding results are present in Figure 4, Figure 5, Figure 6, Figure 7 and Figure 8. Figure 4 showed that the CMCase activity was highest at 60 °C as protein degenerated gradually at a higher temperature. Figure 5 showed that the enzyme kept 90% of activity at 40 °C to 50 °C, the remained CMCase activity decreased obviously when higher than 50 °C. Figure 6 showed that the activity increased sharply from pH = 2.0 to 4.0, when pH is higher than 6.0, the activity decreased, so the optimum reaction pH is 6.0. Figure 7 showed that the CMCase activity of cellulase remained stable at the range of 5.0–6.0, the activity could keep 80%. If pH < 5.0 or pH > 6.0, the CMCase activity decreased, and the cellulase was sensitive to pH. Figure 8 showed that Zn^2+^, Ca^2+^ and Mn^2+^ help cellulase while Co^2+^ and Cu^2+^ inhibit the enzyme activity.

The temperature could speed up the reaction, but the activity of cellulase would fade along with the increasing temperature. The space structure of the enzyme would be destroyed when over an acidic or basic environment, causing the change of conformation, and the loss of enzyme activity. The results showed that the optimum reaction temperature and pH is 60 °C and pH 5. As the component of cellulase is protein, its structure is unique, and metal ions carry more than one positive charge, its effect is stronger than proton. Besides, metal ions have a complexing action, which could maintain the concentration of solution at a stable stage. Therefore, metal ions can clearly affect cellulase activity. The results showed that Zn^2+^, Ca^2+^ and Mn^2+^ are its activators; they can significantly promote its activity. The relative enzyme activity is respectively about 139.2%, 125.5% and 131.4% to the blank. In contrast, Co^2+^ and Cu^2+^ can obviously inhibit its activity. The relative enzyme activity is just 17.6% and 5.9% to the blank, respectively. It also can be seen that the stability of this cellulase is perfect. Even it was incubated at 50 °C for 1 h, the residual activity can maintain it at 90%. Since it was stored in pH 5 buffer for 24 h, its residual activity was about 85%. These enzymatic properties of the newly obtained cellulase are similar to the cellulase from *Bacillus mycoides* S122C [18].

## 4. Discussion and Conclusions

*Trichoderma virens* produced enzyme activity is up to 31.5 U/mg. This is high compared with *Fusarium Oxysporum*, which produced cellulase, and its enzyme activity was only 1.43 IU/mL [19], and *Aspergillus sydowii*, the CMCase of which was 1.32 IU/mL [20].

A novel *Trichoderma virens* was isolated from straw cropland. It can secrete high activity cellulase. The cellulase is a triple-subunit complex with molecular masses of 62, 58 and 16 kDa of each subunit. The optimum temperature and pH of the cellulase are 50 °C and pH 5.0. Zn^2+^, Ca^2+^ and Mn^2+^ could remarkably promote the enzyme activity. Conversely, Cu^2+^ and Co^2+^ are strong inhibitors to the cellulase. It possesses good stability in terms of thermal and pH factors. Based on these findings, it can be concluded that this newly obtained microbe is a good prospect for practical cellulase production and has good application for the new cellulase.

The *Trichoderma virens* ZY-01 was isolated for its high yield of cellulase, which is expected to be useful for hydrolysis of cellulosic and hemicellulosic substrates at proper temperatures, particularly for converting biomass into biofuels, to solve the energy crisis and the environmental pollution problem.

## Figures and Tables

**Table 1:** Isolated strains for cellulase production.

Strain ^1^	Colony Diameter/cm	Hydrolysised Circle Diameter/cm	Colony Color	Colony Morphology
ZY-01	2.30 ± 0.22	4.32 ± 0.26	Early phase white, Anaphase green	Edge irregular dentatus, Surface filiform
ZY-02	1.87 ± 0.41	2.82 ± 0.38	white	Edge irregular dentatus, Surface filiform
ZY-03	2.12 ± 0.49	3.02 ± 0.37	Front milk white, Back yellow	Edge irregular dentatus, Surface filiform
ZY-04	3.27 ± 0.43	fuzzy	Front yellow-white, Back red	Edge irregular dentatus, Surface smooth

^1^ ZY-01: soil sample from wheat field, ZY-02 and ZY-03: soil sample from Yangtze River riverside, ZY-04: soil sample from paper mill.

**Table 2:** Results for purification of cellulose from *T. virens* ZY-01 culture broth.

Procedure	Protein Content/mg	Enzyme Activity/IU	Specific Activity IU/mg	Yield %	Purification Fold
Crude enzyme	197.3	174.1	0.88	–	–
Fractional precipitation	21.6	135.3	6.26	77.7	7.11
Anion exchange chromatography	15.8	106.4	6.73	61.1	7.65
Gel chromatography	2.6	81.9	31.5	47.04	35.8
